# A Case of Pulmonary Tuberculosis, Severe Dengue, and COVID-19 With Venous Thromboembolism in an Indian Male: World's First Case

**DOI:** 10.7759/cureus.27537

**Published:** 2022-07-31

**Authors:** Sankalp Yadav

**Affiliations:** 1 Medicine, Shri Madan Lal Khurana Chest Clinic, Moti Nagar, New Delhi, IND

**Keywords:** deep vein thrombosis (dvt), pulmonary thromboembolism, dengue, covid-19, tuberculosis

## Abstract

The pandemic of coronavirus disease 2019 (COVID-19) has resulted in extensive morbidity and mortality. Not only the viral pandemic has affected almost every single person on this planet directly or indirectly but it has resulted in reporting of several cases with rare presentations of common diseases. The present case is a rare presentation wherein COVID-19 with pulmonary tuberculosis (TB), severe dengue, and venous thromboembolism was diagnosed in an Indian male. This case is crucial as no such case has ever been reported in the literature. Also, during the pandemic other diseases received relatively less attention. Therefore, establishing the diagnosis in a situation where the health systems were oversaturated was a difficult task, and thus present case would be an important addition to the literature. Further, diseases like COVID-19, TB, and dengue share many similarities like pathogenesis, clinical features, and lab results, therefore, this case would help the clinicians, especially in settings where these diseases are common.

## Introduction

The pandemic caused by the severe acute respiratory syndrome coronavirus 2 (SARS-CoV-2) i.e., coronavirus disease 2019 (COVID-19) has led to large-scale morbidity and mortality [1]. This viral pandemic which started in Wuhan, China in 2019 has spared no gender or ethnicity [2]. To date, there are 574,834,831 cases of COVID-19 with 6,402,668 fatalities [3]. The major contributor to this persisting pandemic is the rapid mutations in the viral ribonucleic acid (RNA) [4]. The development of COVID-19 vaccines has contributed to a great deal in reducing the mortalities yet the cases are regularly reported. This could be due to multiple factors like lack of use of proper protective measures including social distancing, a false feeling of being protected from COVID-19 due to vaccination, reduced/no use of sanitizers, etc.

Tuberculosis (TB) is an infectious disease known for ages and is caused by *Mycobacterium tuberculosis *(MTB). It is a noteworthy contributor to morbidity and mortality [5]. In high-burden countries like India TB prevalence and incidence is very high. Furthermore, the risk of drug-resistant TB is also very high in these densely populated countries.

Dengue is a viral infection transmitted by the bite of infected mosquitoes [6]. The mosquitoes that are the vectors for transmitting the disease are female *Aedes aegypti *mosquitoes and, to some degree, *Aedes albopictus* [6]. The disease is caused by dengue virus (DENV) which has four serotypes with the possibility to infect four times [6]. Dengue is common in tropical and sub-tropical climates worldwide [6]. Most of the time Dengue is a subclinical disease however it could also present as severe flu-like symptoms [6].

In this present case author report, a rare presentation of COVID-19 with pulmonary TB, severe dengue, and venous thromboembolism (VTE) was diagnosed in an Indian male. This case is unique as coetaneous infections of dengue with COVID-19 and pulmonary TB with VTE have never been reported in the literature. These diseases especially pulmonary TB, COVID-19, and dengue have certain similar clinical features and therefore it requires a high index of suspicion backed with a detailed laboratory work-up to diagnose these infections together. Another aspect that makes this case interesting is the fact that the diagnosis and management of diseases with overlapping signs and symptoms and laboratory parameters in the background of an ongoing pandemic where the health facilities were oversaturated was a remarkable achievement.

## Case presentation

A 23-year-old Indian male student belonging to middle-class family came as a referred case with chief complaints of fever with chills for 14 days, cough with expectoration for two weeks, breathlessness for two weeks, night sweats for two weeks, loose motion for two days, loss of appetite for one week and pain with swelling in right lower limb for three days and retro-orbital pain for two days. He also had one episode of bleeding from nose.

He was apparently well two weeks back when he developed fever with chills without rigor, it was evening rise in pattern associated with night sweats and was relieved after taking over-the-counter antipyretic (paracetamol). He also had cough which was associated with a thick, yellow-colored, non-foul smelling expectoration for two weeks. Coughing increased on walking and subsided after taking an expectorant (ambroxol hydrochloride). He had breathlessness on exertion and was relieved by rest. Also, he had six episodes of loose motion for two days. The consistency was watery, foul-smelling, yellow-colored, non-blood tinged. In addition, he had a loss of appetite for one week with no remarkable weight loss. And he had pain with swelling in the right lower limb for three days. The swelling was present over the whole right lower limb, and began after a cramp in the right calf was amalgamated with mild erythema and edema. One episode of nose bleeding was reported which lasted for about two minutes.

He was a non-smoker with no history of substance abuse. There was no history of major medical or surgical intervention in the past. Further, there was no history of headache, nausea, vomiting, bleeding, rash, or any trauma. Besides, there was no history of TB or COVID-19 in him or any of his close contacts. And he was a non-immigrant with no history of unemployment, imprisonment, or contact with commercial sex workers or drug dealers. He was not vaccinated against COVID-19.

General examination revealed a febrile patient (temperature 101 degrees Fahrenheit), pulse 111 per minute, blood pressure 122/80 mmHg, oxygen saturation (SpO2) 93% on room air. Local examination of the right lower limb revealed a generalized swelling with mild erythema, warm to touch, and non-pitting in nature. It was associated with pain and the Homans sign was positive.

Systemic examination was noteworthy for a dull note on percussion on the left hemithorax with diminished vocal repercussions. On auscultation, there was crepitation on the upper and lower lobes of the left lung. The rest of the systemic examination was unremarkable.

**Figure 1:**
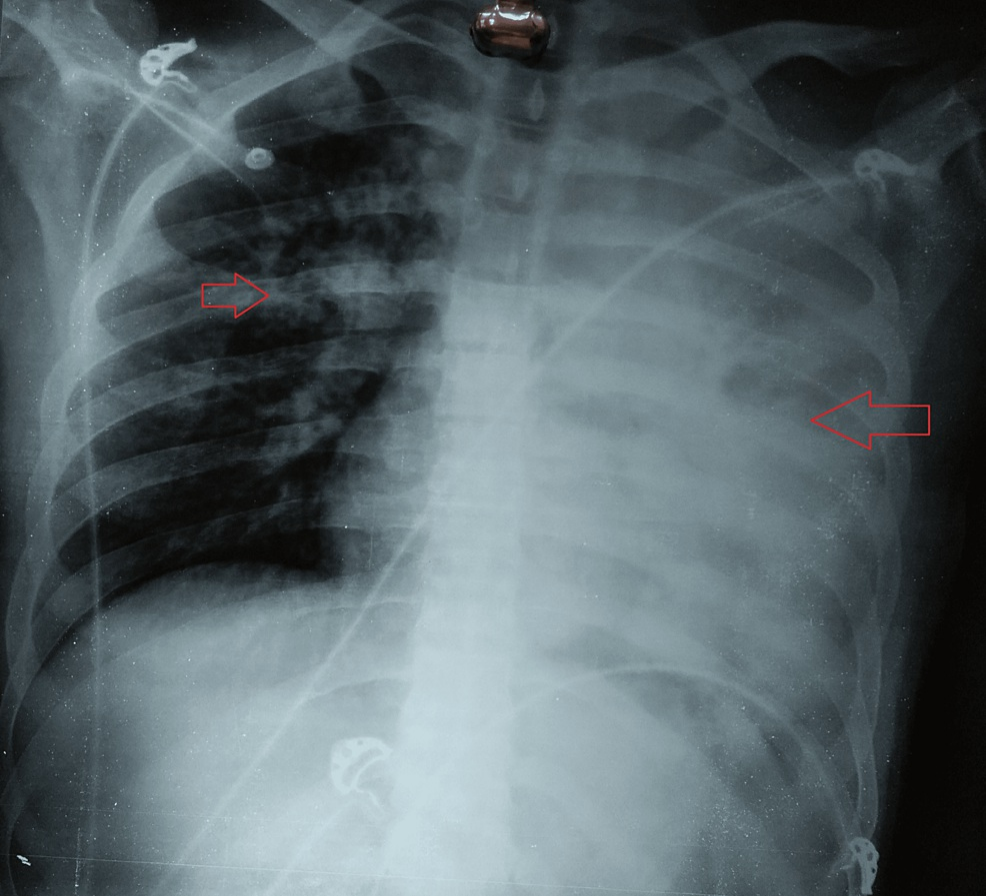
Chest radiograph (P-A view) showing consolidation on the left upper and lower lobes and right upper lobe

A provisional diagnosis of pulmonary TB with acute gastroenteritis was made and the patient was referred to the lab for detailed investigations (Table 1). An on-spot rapid antigen test for SARS-CoV-2 was done which was positive. Other investigations like sputum for acid-fast bacilli (AFB) test and a cartridge-based nucleic acid amplification test (CBNAAT) of the sputum were suggestive of *Mycobacterium tuberculosis *detected (low) and were sensitive to rifampicin. Dengue serology showed positive IgM antibodies with a value of 1.90 on enzyme-linked immunosorbent assay (ELISA). Complete blood count revealed normocytic, normochromic anemia with neutrophilia, a total platelet count of 80000/mcL, a raised hematocrit, and a raised erythrocyte sedimentation rate. Liver function tests were remarkable for raised aspartate aminotransferase, alanine aminotransferase, and alkaline phosphatase. ECG was unremarkable. Chest radiograph (P-A view) was suggestive of consolidation on the left upper and lower lobes and right upper lobe (Figure 1).

**Table 1:** Investigations HGB: Hemoglobin; PLT: Platelets; WBC: White Blood Cell; DLC: Differential Leukocyte Count MCV: Mean Corpuscular Volume; MCH: Mean Corpuscular Hemoglobin; MCHC: Mean Corpuscular Hemoglobin Concentration; RDW: Red Cell Distribution Width; PCV: Packed cell volume; AST: Aspartate Aminotransferase; ALT: Alanine Aminotransferase; ALK PHOS: Alkaline Phosphatase; GFR: Glomerular Filtration Ratio; USG-WA: Ultrasonography-whole abdomen

Investigation	Results		Reference range
	Admission	Discharge	
HGB	11.0	11.6	11.5-16.0 g/dL
MCH	30.7	31.0	27-33 pcg
MCHC	33.1	33.0	31-36 g/dL
MCV	92.1	92.0	85-100 fl
PCV	39.0	39.0	38.3% to 48.6%
RDW	13.2	13.0	0-14%
RBC	4.7	4.8	4.7 to 6.1 million cells/mcL
WBC	10.3	11.0	4.5-12.0 K/uL
DLC-			
Neutrophils	76	75	55-70%
Lymphocytes	12	12	20-40%
Monocytes	10	10	2-8%
Eosinophils	1	2	1-4%
Basophils	1	1	0-1%
ESR	90.0	78.0	0 to 22 mm/hr
Serum sodium	131.0	132.0	135/145 mmol/L
Serum potassium	4.65	4.60	3.5-5.1 mmol/L
Serum calcium	8.10	8.11	8.5-10.5mmol/L
Serum chloride	98.0	99.0	98-107 mmol/L
Blood culture	Sterile	Sterile	Sterile
Dengue serology IgG antibody	Negative	Negative	Positive-Negative
Serum bilirubin (total)	0.61	0.7	0.2-1.0 mg/dL
Serum bilirubin (direct)	0.37	0.5	0.2-1.0 mg/dL
Serum bilirubin (indirect)	0.24	0.3	0.2-1.0 mg/dL
ALK PHOS	120.0	80.0	30-115u/L
ALBUMIN	3.4	3.3	3.5-5 g/dl
Serum creatinine	0.59	0.8	0.51-0.95 mg/dL
AST	129.0	38.0	0-40u/L
ALT	133.0	40.0	0-40u/L
GFR	62.0	66.0	>60 /min
D-Dimer	0.8	0.3	< 0.4 μ/mL
Serum ferritin	291	300	12 to 300 ng/mL
Serum lactate dehydrogenase	301	290	140-280 U/L
Anti HCV antibodies	Non-reactive	Non-reactive	Reactive-Non-reactive
HIV	Non-reactive	Non-reactive	Reactive-Non-reactive
Fasting blood sugar	90.0	99.0	70-99 mg/dL
Activated partial thromboplastin time	33	29	25-35 seconds
2D-Echocardiography	Left ventricular ejection fraction-54%		
USG-WA	WNL	WNL	

**Figure 2:**
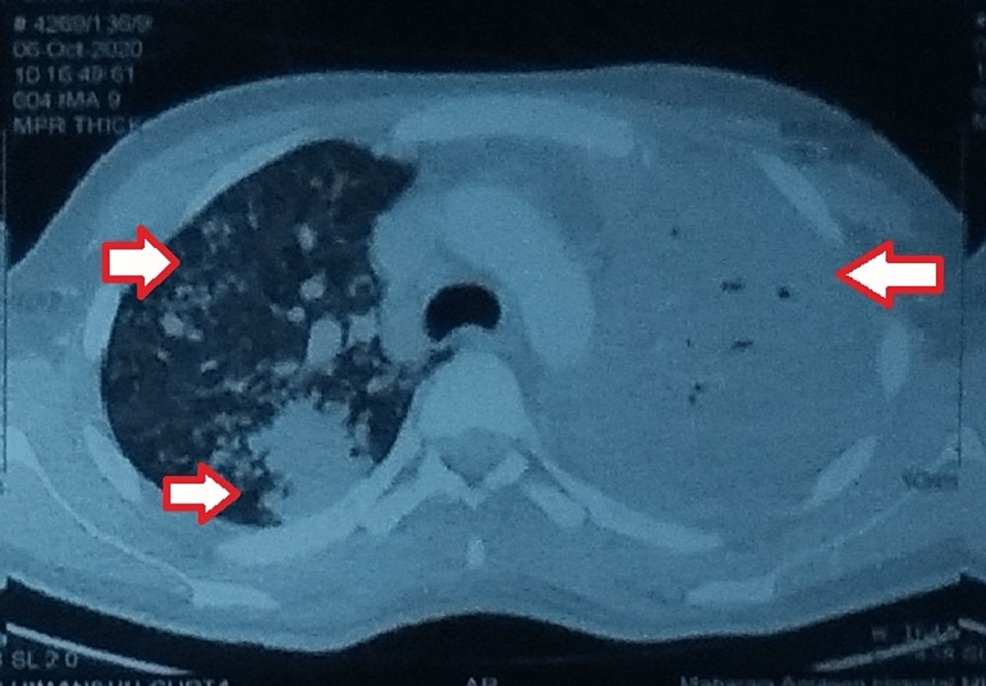
Contrast-enhanced computed tomography of the chest (CECT-chest) showing bilateral involvement

A venous color Doppler of lower limbs indicated external iliac, common femoral, superficial femoral, and popliteal vein thrombosis on the right side. A bedside ultrasound of the chest revealed mild left-sided pleural effusion with basal collapse/consolidation. A contrast-enhanced computed tomography of the chest (CECT-chest) was suggestive of mild volume loss on the left side with an ipsilateral mediastinal shift. There was consolidation with cavitation on the left upper and lower lobes. Focal right upper lobe consolidations with multiple tree-in-bud nodules. And there was mild left pleural effusion with ill-defined filling defects in the right pulmonary artery. Other notable findings were of mediastinal (prevascular- 1.4 X 1.2 mm) and axillary lymph (3.5 X 2.5 mm) nodes (Figure 2).

A CT-pulmonary angiography was done which showed pulmonary thrombosis involving right lower lobe branches with extension into segmental arteries. Other relevant investigations are mentioned in Table 1. The acquired thrombophilia was ruled out with no history of COVID-19 and vaccination against COVID-19.

Finally, a diagnosis of pulmonary TB with severe dengue, and venous thromboembolism (VTE) in a COVID-19-positive male was made. Management was started with intravenous (IV) normal saline and ringers lactate alternately at the rate of 80 ml/hour, tablet paracetamol 650 mg two times a day, a probiotic capsule lyophilized *Saccharomyces boulardii *250 mg twice daily. For COVID-19 hydroxychloroquine 400 mg loading dose followed by 200 mg twice daily for a seven-day course, inhalational budesonide twice daily, vitamin B-complex once daily, vitamin C 500 mg three times a day, ursodeoxycholic acid 300 mg twice a day, and betadine gargles twice daily. Anti-tuberculosis (anti-TB) medications were started with fixed-dose combinations of rifampicin (450 mg), isoniazid (300 mg), ethambutol (1000 mg), and pyrazinamide (1200 mg). The management of VTE involved twice daily administration of subcutaneous enoxaparin 40 mg twice daily with strict activated partial thromboplastin time (aPTT). After seven days of admission, his condition improved. There was relief in pain in the lower limb and swelling was reduced. There were a few episodes of fever and cough. And the liver function test returned to normal with a platelet count of 110000/mcL. On his request, he was discharged on nicoumalone, anti-TB medications, doxofylline, nebulization with budesonide, levosalbutamol and ipratropium inhaler, paracetamol, pantoprazole, vitamin B-complex, vitamin C, betadine gargles, ursodeoxycholic acid, and a protein supplement. He was advised breathing exercises and yoga and referred to the department of Surgery for VTE and was counseled to continue his anti-TB medications with a timely follow-up in the OPD however, the patient was lost to follow up.

## Discussion

Diseases like COVID-19, pulmonary TB, and dengue have overlapping pathogenesis, clinical features, and lab results which are widely available in literature [5,7,8]. Prompt diagnosis and management of cases with these infections require a high degree of suspicion and lab investigations [5]. The delay could have life-threatening consequences for the patients [7].

The present case is unique with all three diseases i.e., COVID-19, pulmonary TB, and severe dengue with VTE in a young man. This case emphasizes the importance of paying attention to other diseases, especially during the current pandemic. As mentioned earlier, diseases like dengue could be asymptomatic but in severe cases, there is a very high risk of life [6]. There could be complications due to severe bleeding, organ impairment, and/or plasma leakage [6]. Reports of dengue with COVID-19 are available widely and the data suggests there is a higher fatality risk in dengue with concomitant COVID-19 [9].

Isolated reports of TB with COVID-19 are available in the literature mostly as case reports [5]. These cases have been reported not only in drug-sensitive TB but drug resistant-TB as well [5]. Data regarding the association between TB and COVID-19 is lacking but from the available reports, and till the reports of large scale studies are available, it could be presumed that both these infections could occur simultaneously and immediate management is essential.

Further, pulmonary thromboembolism (PTE) is known to be associated with TB and other infections [10]. PTE is a life-threatening condition and requires immediate management [10]. The incidence of PTE is not exactly known but in a recent study by Di Bari et al., it was recommended that PTE risk assessment should be done in cases of TB [10]. In another retrospective analysis, out of 7905 patients diagnosed with TB, 0.6% exhibited PTE, deep venous thrombosis (DVT), or both i.e., VTE on or after the diagnosis of TB [10]. Hypercoagulability, venous stasis, and endothelial dysfunction constituting Virchow's triad could be the possible causes of VTE in TB [11].

Other important contributors could be TB’s thrombogenic state which encompasses reactive thrombocytosis, anemia, and the release of pro‐inflammatory cytokines damaging the vascular endothelium during the disease process [11]. Another notable point for a hypercoagulable state is the imbalance in the pro‐coagulant and anticoagulant factors such as increased fibrinogen, factor VIII plasminogen activator inhibitor 1 plasma levels, and depressed anti‐thrombin III and protein C levels in the initial months of management for TB [11]. However, due to oversaturated labs and lack of free slots during the third wave of COVID-19 these tests were not done in the present case.

## Conclusions

Diseases like TB, COVID-19, and dengue have been known to have a high number of similarities. The situation becomes even more alarming when complications like VTE are also detected. This case highlights the occurrence of the three diseases in a male with no significant history. Moreover, the case was difficult to treat due to the poor financial situation of the patient, overwhelmed health facilities, lockdowns, and social and psychological impact of COVID-19 on the patient. Still, this patient reported well on time and was managed conservatively. The only limitation of this presentation is that the patient was lost to follow-up and thus his present condition could not be assessed.
